# Optimization of echinococcosis control measures based on system dynamics

**DOI:** 10.1371/journal.pcbi.1013186

**Published:** 2025-09-30

**Authors:** Jinrui Ma, Xingyu Liu, Zengwen He, Haohao Zhang, Yonghui Ma, Ziqiu Fan, Xuehui Zhao, Ji Zhi, Qing Cao, Huiwen Xue, Huitian Gou

**Affiliations:** 1 Department of Veterinary Public Health, College of Veterinary Medicine, Gansu Agricultural University, Lanzhou, China; 2 Department of Applied Statistics, College of Science, Gansu Agricultural University, Lanzhou, China; Animal and Plant Health Inspection Service, UNITED STATES OF AMERICA

## Abstract

This study aimed to provide strategies to optimize the prevention and control measures of echinococcosis in Gansu Province. Based on the transmission mechanism of echinococcosis, a System Dynamics (SD) model of echinococcosis in Gansu Province was established using Vensim software. Through this model, parameters such as vaccination coverage, domestic dog deworming coverage, slaughter management level, and health education level were adjusted to predict the changing trends in the number of human cases and infected sheep. The results showed that the number of human cases was most sensitive to the adjustment of health education level, followed by changes to vaccination coverage. The number of infected sheep was most sensitive to the adjustment of vaccination coverage, followed by the adjustment of slaughter management level. Based on these simulation results, the prevention and control measures for echinococcosis in Gansu Province were further optimized. The results showed that if the health education level and the vaccination coverage were increased by 50% and 40%, respectively, based on the current prevention and control levels, the number of human cases would approach zero by approximately 2026. When the vaccination coverage and slaughter management level increased by 60% and 45%, respectively, based on current prevention and control levels, the number of infected sheep would approach zero by approximately 2027. This study applied the SD method to the prevention and control of echinococcosis for the first time, and the results can provide key decision-making recommendations to optimize the prevention and control measures of echinococcosis in Gansu province.

## Author summary

Echinococcosis is a prevalent disease in Gansu Province, China. Due to factors such as delayed economic development, complex geographical conditions, and diverse religious practices, controlling this disease poses significant challenges. In this study, we used a system dynamics approach and employed the Vensim simulation software to build an echinococcosis model for Gansu Province. By adjusting key parameters in the model, we explored the effects of different control measures. This research aims to optimize echinococcosis control strategies in Gansu and provide scientific evidence for policymakers. We input historical data on the number of human cases and infected sheep from 2015 to 2022 into the model for validation, and the results showed a high degree of agreement between the simulated and actual values, confirming the model’s validity. We then simulated nine scenarios by adjusting four key parameters: vaccination coverage, domestic dog deworming coverage, slaughter management level, health education level. The results indicated that human publicity and education and sheep slaughtering management are the most crucial factors in controlling human and sheep echinococcosis, respectively. If health education level and vaccination coverage increase by 50% and 40%, respectively, human echinococcosis could be effectively controlled by 2026. Similarly, if vaccination coverage and slaughter management level improve by 60% and 45%, respectively, sheep echinococcosis could be effectively controlled by 2027.

## 1. Introduction

Echinococcosis, also known as hydatid disease, is a zoonotic parasitic disease caused by *Echinococcus* tapeworms at the larval stage, and poses serious threats to human health and economic development [1,2]. China is among the countries most severely affected by echinococcosis, primarily in the western pastoral and semi-pastoral regions, affecting 370 counties across the Inner Mongolia, Yunnan, Tibet, Sichuan, Gansu, Qinghai, Ningxia, Xinjiang, and Shaanxi provinces and autonomous regions [3–5]. According to statistics, about 380,000 people nationwide are infected with echinococcosis, and 50 million people are at risk of infection [6]. Echinococcosis has a significant economic impact on livestock farming, leading to losses due to livestock death, organ discards, and reduced productivity [7]. The disease causes significant suffering and imposes a heavy economic burden on families, contributing to poverty in the western pastoral regions [8–11].

The life cycle of *Echinococcus* species comprises three distinct stages: the egg, the larval stage, and the adult stage. The adult cestodes reside in the small intestine of definitive hosts, which are primarily canids such as domestic dogs, wolves, and foxes. Gravid proglottids and eggs are shed in the feces of definitive hosts, leading to environmental contamination of pastures, water sources, and domestic areas, or adherence to the fur of animals. Intermediate hosts, including livestock (e.g., sheep, cattle, and pigs), become infected upon ingestion of the eggs. Within the small intestine of the intermediate host, the eggs hatch, releasing oncospheres that penetrate the intestinal mucosa and migrate via the bloodstream to target organs, particularly the liver and lungs. Over a period of 3–5 months, the oncospheres develop into hydatid cysts. When definitive hosts ingest tissues containing these hydatid cysts, the protoscoleces within the cysts develop into adult tapeworms in the small intestine, completing the life cycle. Humans are accidental intermediate hosts and become infected exclusively through the ingestion of eggs; they do not play a role in the continuation of the parasite’s life cycle [12–14].

System Dynamics (SD) is a discipline based on feedback, control, information, and nonlinear systems theories. It employs computer simulations to study feedback systems and predict future trends [15]. The SD approach helps model the interactions among various factors in a system and is particularly useful for understanding complex systems, such as disease transmission [16]. By simulating these interactions, SD models can provide valuable insights into effective management strategies, particularly for infectious disease control [17].

For echinococcosis, large-scale experimental studies are often impractical, making computer-based simulations essential tools for understanding disease transmission. SD models enable the analysis of various control measures such as dog deworming and livestock vaccination by simulating their effects on disease transmission [18]. This approach allows the optimization of prevention and control strategies in a safe and controlled environment [19].

Gansu Province, one of the most severely affected regions in China, faces significant challenges in controlling echinococcosis owing to its complex geographical landscape, diverse religious customs, and limited public health resources. Low educational levels in these ethnic minority areas contribute to limited awareness of echinococcosis among local herders, leading to high disease prevalence [20]. Many dogs in these regions excrete feces containing echinococcosis eggs, contaminating water sources, soil, and grasslands, which further increases the risk of transmission [3]. These factors have resulted in high echinococcosis incidence rates in Maqu, Tianzhu, and Subei counties, severely impacting local economies and public health. These challenges are compounded by the high number of animal hosts and wide distribution of infection sources [21]. Therefore, achieving optimal prevention and control of echinococcosis with limited resources is a crucial problem requiring urgent resolution. The present study aimed to simulate and optimize key parameters such as vaccination coverage, domestic dog deworming coverage, slaughter management level, and health education level using Vensim software to establish an SD model according to the transmission mechanism of echinococcosis. The goal was to explore the feedback mechanisms of different parameter values on the comprehensive prevention and control of echinococcosis, optimize existing prevention and control measures, reasonably allocate limited resources, and provide recommendations for the prevention and control of echinococcosis in Gansu Province, thereby ensuring safety of human life and promoting the healthy development of animal husbandry.

## 2. Methods

### 2.1. Study area

**Fig 1:**
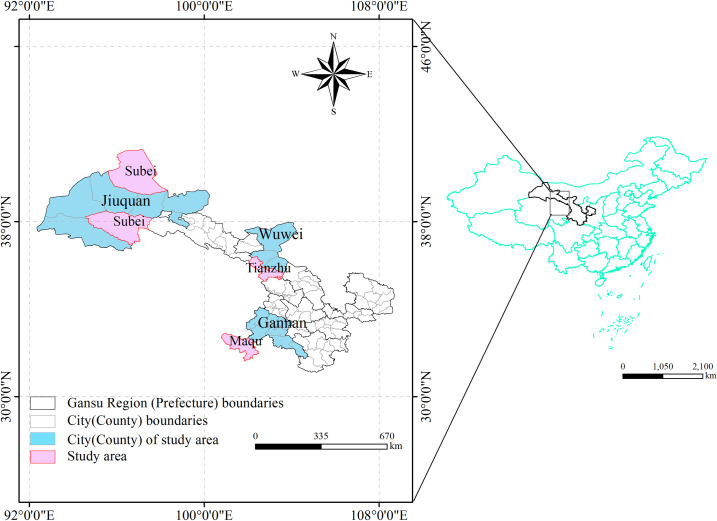
Overview of the study area. Audit number is GS(2023)2767, no modifications to the base map, URL:http://bzdt.ch.mnr.gov.cn/index.html. This diagram was created by the software GIS 10.6,URL:https://desk-top.arcgis.com/zh-cn/index.html.

Maqu County (located between latitudes 33°06′30″ and 34°30′15″ North, and longitudes 100°45′45″ and 102°29′00″ East), Tianzhu County (between latitudes 36°31′ and 37°55′ North, and longitudes 102°07′ and 103°46′ East), and Subei County (between latitudes 38°13′ and 40°1′ North, and longitudes 94°33′ and 98°59′ East) are pastoral regions in Gansu Province, characterized by vast grasslands primarily used for cattle and sheep farming (Fig 1).

### 2.2. Establishment of an SD model for echinococcosis epidemic and control

#### 2.2.1. Formulating a dynamic assumption.

A dynamic assumption is the theory of a structure that generates a reference pattern. These assumptions can be expressed verbally as causal loop diagrams or stock and flow diagrams. Formulating a dynamic assumption helps determine the elements to include and exclude from the model [22]. To highlight the main focus of this study, define the model boundaries, reduce complexity of the simulation model, and clarify the internal logical relationships within the model, the following assumptions are proposed:

**Assumption 1** The definitive host, dogs, can only come into contact with the organs of intermediate host animals, specifically sheep, and not with those of other intermediate host animals, such as cattle and pigs.

**Assumption 2** This model focuses solely on sheep within Gansu Province and does not consider the risk of echinococcosis transmission from the movement of sheep from other provinces.

**Assumption 3** The infectivity of *Echinococcus* eggs remains constant and is unaffected by changes in natural environmental factors such as temperature, humidity, light, wind speed, and air pressure. Each egg has the same infectivity in intermediate hosts, humans, and sheep.

#### 2.2.2. Construction of the system stock-flow model.

**Fig 2:**
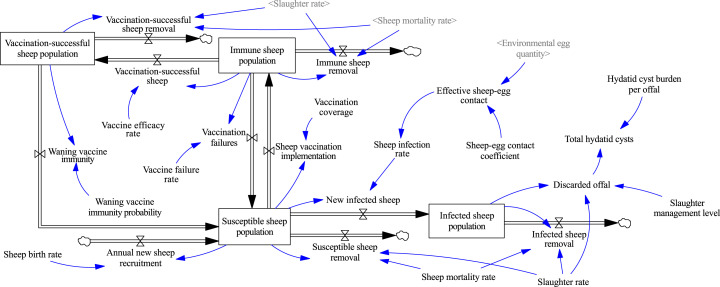
Sheep echinococcosis stock-flow diagrams.

**Fig 3:**
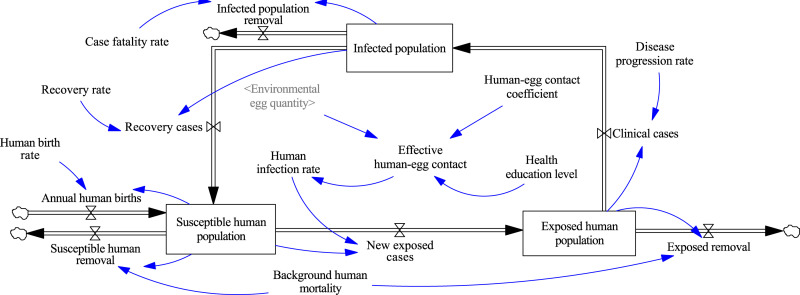
Human echinococcosis stock-flow diagrams.

**Fig 4:**
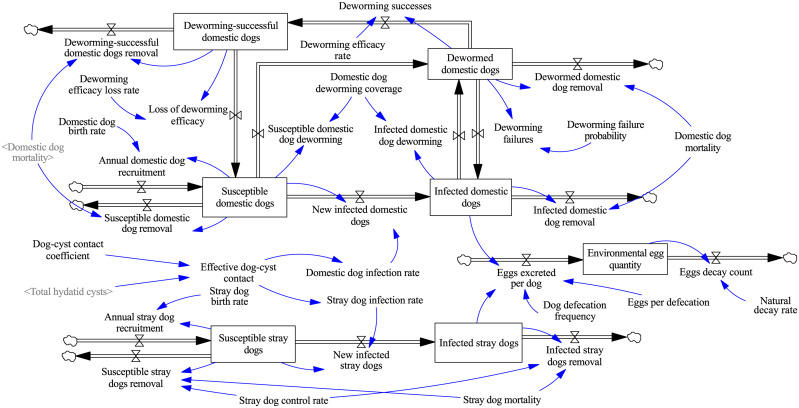
Dog echinococcosis stock-flow diagrams.

In Gansu Province, the predominant form of echinococcosis is cystic echinococcosis, which spreads through the parasitization of *Echinococcus* tapeworms in intermediate hosts (humans and sheep) and definitive hosts (dogs, including both domestic and stray dogs). Factors such as the scale of sheep farming, the range of dog activities, and human living customs can exacerbate the spread of echinococcosis. Meanwhile, government agencies have been implementing control measures, such as deworming domestic dogs, vaccinating sheep, managing sheep slaughter, and educating the public to mitigate the prevalence of the disease. To construct an SD stock-flow model for the transmission and control of echinococcosis in Gansu Province, we first determined the system boundaries and feedback relationships among the key variables and provided detailed information about the causal feedback loops in S1 Text. This model includes the sheep echinococcosis stock-flow model (Fig 2), human echinococcosis stock-flow model (Fig 3), and dog echinococcosis stock-flow model (Fig 4).

#### 2.2.3. Setting key variables and parameter values.

The variables used to construct the SD model were categorized into basic and parametric data and the equations for all variables are provided in S2 Text. Basic data included the susceptible sheep population, susceptible human population, and number of infected individuals. These data were sourced from the Gansu Province Bureau of Statistics (Statistical Yearbooks 2015–2022) (https://tjj.gansu.gov.cn/) and Health Commission of Gansu Province(https://wsjk.gansu.gov.cn/). Parametric data, such as the effective contact coefficient between sheep and *Echinococcus* eggs, effective contact coefficient between dogs and cysts, sheep mortality, dog mortality, human infection mortality, and natural human mortality rates, were derived from relevant literature. The specific parameter settings are listed in Table 1.

**Table 1:** Main parameter data settings.

Parameter	Parameter value	Source
Sheep-egg contact coefficient	1.08 × 10^-9^	[23]
Sheep birth rate	0.4146	[24]
Slaughter rate	0.2	[23]
Sheep mortality rate	0.152	[23]
Vaccine failure rate	0.67	[23]
Dog-cyst contact coefficient	7.1 × 10^-8^	[23]
Domestic dog birth rate	0.3212	[24]
Stray dog birth rate	0.3212	[24]
Domestic dog mortality	0.08	[25]
Stray dog control rate	0.4	[23]
Stray dog mortality	0.08	[25]
Deworming failure probability	0.4	[23]
Human-egg contact coefficient	1.004 × 10^-12^	[23]
Human birth rate	0.0146	[25]
Disease progression rate	1/14	[25]
Case fatality rate	0.022	[23]
Recovery rate	0.75	[23]
Background human mortality	0.01363	[18]
Vaccine efficacy rate	0.97	[26]
Deworming efficacy rate	0.9	[27]

### 2.3. Validation of the SD model for echinococcosis epidemic and control

#### 2.3.1. Structured validation of the SD model.

Based on a comprehensive review of the literature on the epidemiological status and control measures for echinococcosis in Gansu Province as well as field surveys conducted in endemic areas, timely modifications were made to address the deficiencies in the constructed model. After completing the overall model construction, the structural relationships among the variables were re-examined to ensure that the model accurately reflected the real situation. Finally, the “Check Model” and “Units Check” functions in Vensim PLE 10.0.0 software were used to validate the overall system structure and units.

#### 2.3.2. Historical validation of the SD model.

To verify whether the model could accurately reflect the actual control measures, real data on infected sheep and the number of human cases from 2015 to 2022 were selected as historical values for model validation. The fit between the historical values and simulation results was then compared.

### 2.4. Scenario design

Key control measures, such as vaccination coverage, domestic dog deworming coverage, slaughter management level, and health education level, were selected as input variables, while the number of human cases and infected sheep were chosen as output variables. Using the current control measures (Current scenario) as the baseline, the calculation of the baseline value is in S3 Text. The control measures currently implemented in Gansu Province were converted into input variable values. To isolate the effect of modifying each control measure, each of the four input variables was adjusted by 30% to simulate the changes in the control measures while the values of other variables were kept constant. This resulted in a total of nine control scenarios, designated as Scenarios 1–1~4–2, along with the current scenario. The specific design schemes are listed in Table 2.

**Table 2:** Parameter design of echinococcosis control measures in gansu province.

Protective measures	Baseline values	Direction	Scenarios
Vaccination coverage	0.1996	0.2594 (+30%)	Scenario 1–1
		0.1397 (-30%)	Scenario 1–2
Domestic dog deworming coverage	0.6758	0.8785 (+30%)	Scenario 2–1
		0.4731 (-30%)	Scenario 2–2
Slaughter management level	0.5152	0.6698 (+30%)	Scenario 3–1
		0.3606 (-30%)	Scenario 3–2
Health education level	0.6542	0.8505 (+30%)	Scenario 4–1
		0.4579 (-30%)	Scenario 4–2

The plus sign (+) indicates an increase, and the minus sign (-) indicates a decrease in the respective parameter.

### 2.5. Simulation analysis

The system simulation was conducted using Vensim PLE 10.0.0 software. The simulation period spanned from 2015 to 2030 with a one-year time step. The unit of time was set to “Year.” Using the Current scenario as the baseline, scenarios 1–1~4–2 were simulated sequentially. The parameters for vaccination coverage, domestic dog deworming coverage, slaughter management level, and health education level were adjusted in different scenarios. Predicted trends in the numbers of human cases and infected sheep were provides reference strategies for optimizing echinococcosis control measures in Gansu Province.

## 3. Results

### 3.1. Results of SD model validation

#### 3.1.1. Results of structural validation.

Using the “Check Model” and “Units Check” functions in Vensim PLE 10.0.0 software, the overall structure and units of the model were examined. The results indicate that the model structure is sound and ready for further validation.

#### 3.1.2. Results of historical validation.

**Fig 5:**
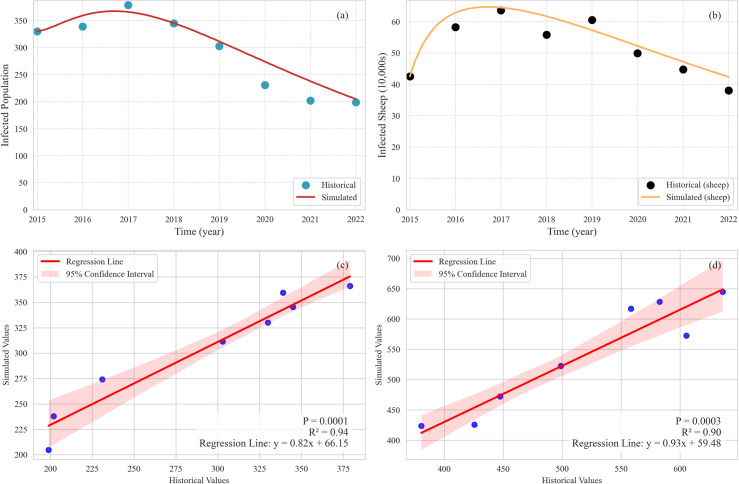
Validation results of historical values. (a) Simulated and historical values of the number of human cases; (b) Simulated and historical values of the number of infected sheep; (c) the relationship between the simulated and historical values of the number of human cases; (d) the relationship between the simulated and historical values of the number of infected sheep. The blue dots represent the correspondence between simulated values and historical values.

The validation results for the historical values of the number of human cases and infected sheep are shown in Fig 5a and 5b. The simulated values for the number of human cases and infected sheep from 2015 to 2022 closely matched the historical values, further confirming the validity of the constructed model and its equations. Regression analyses with 95% confidence intervals further quantified this agreement. For human cases (Fig 5c), the regression line exhibited a tight fit (R2 = 0.94, P = 0.0001), with confidence intervals indicating minimal dispersion around the trend. Similarly, for infected sheep (Fig 5d), the regression line showed strong consistency(R2 = 0.90, P = 0.0003), sup-ported by narrow confidence intervals. These results demonstrate the model’s accuracy in replicating historical trends.

### 3.2 Simulation results of SD model

#### 3.2.1. Simulation results of human cases.

**Fig 6:**
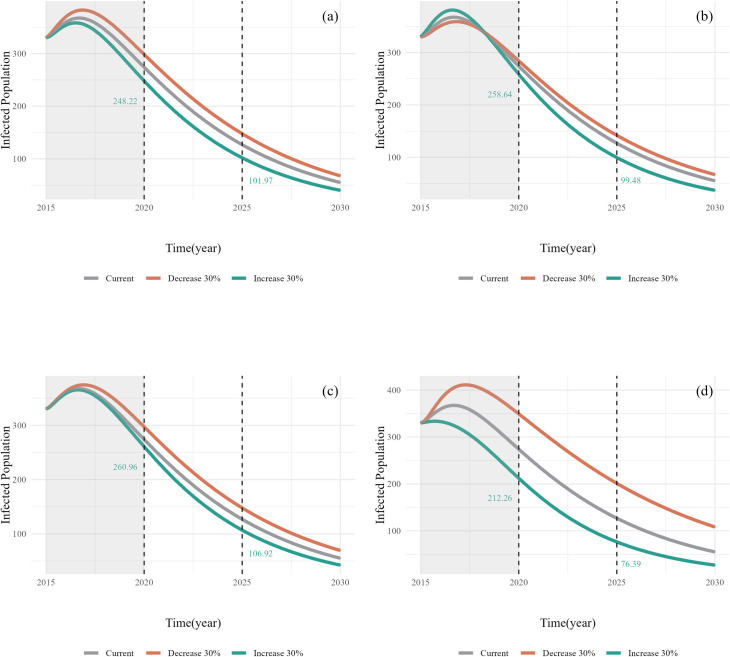
Simulated results of the SD model for the number of human cases under different scenarios. (a) Scenario 1-1/2, variation in vaccination coverage; (b) Scenario 2-1/2, variation in domestic dog deworming coverage; (c) Scenario 3-1/2, variation in Slaughter management level; (d) Scenario 4-1/2, variation in health education level (see Table 2 for a description of the different scenarios).

The simulation results for the number of human cases are shown in Fig 6. In all scenarios with positive control measures, the number of human cases decreased. Scenario 4–1 (enhanced human publicity and education) was the most effective, followed by scenario 1–1 (increased vaccination coverage). In scenario 2–1 (increased domestic dog deworming coverage), the number of human cases initially increased above the current scenario and then decreased to below the current scenario. Conversely, scenario 2–2 (decreased domestic dog deworming coverage) exhibited the opposite trend. Additionally, strengthening sheep slaughter management (Scenario 3–1) and weakening it (Scenario 3–2) resulted in a decrease and increase in the number of human cases, respectively, although these changes were not significant.

#### 3.2.2. Simulation results of infected sheep.

**Fig 7:**
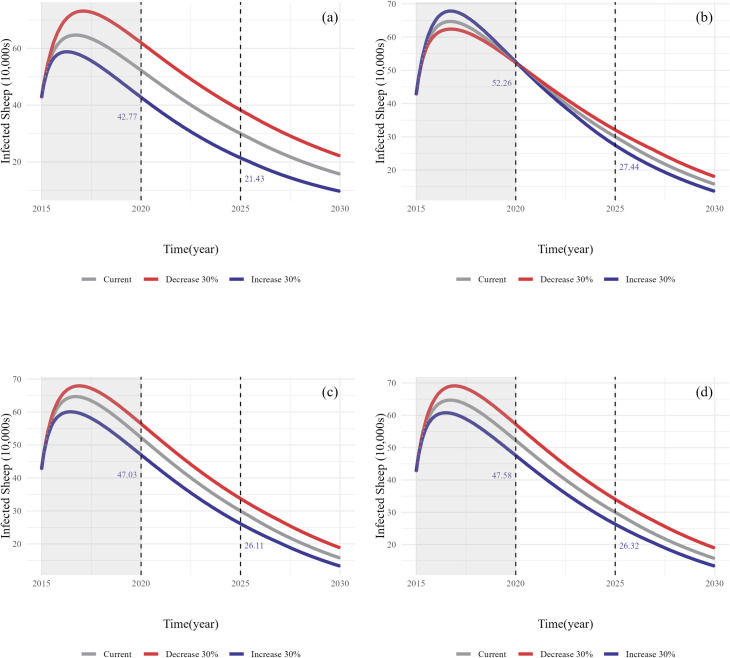
Simulated results of the SD model for the number of infected sheep under different scenarios. (a) Scenario 1-1/2, variation in vaccination coverage; (b) Scenario 2-1/2, variation in domestic dog deworming coverage; (c) Scenario 3-1/2, variation in slaughter management level; (d) Scenario 4-1/2, variation in health education level (see Table 2 for a description of the different scenarios).

As shown in the simulation results for the number of infected sheep in Fig 7, the increase (Scenario 1–1) and decrease (Scenario 1–2) in the vaccination coverage had the most significant impact on the number of infected sheep. Because sheep are the primary intermediate hosts, the trends in the number of infected sheep following the increase (Scenario 2–1) and decrease (Scenario 2–2) in domestic dog deworming coverage were similar to those observed in the number of human cases. Strengthening (Scenario 3–1, Scenario 4–1) and weakening (Scenario 3–2, Scenario 4–2) slaughter management level and health education level resulted in corresponding decreases and increases in the number of infected sheep, respectively. The slaughter management level indicated a slightly greater effect than health education level.

### 3.3. Simulation results of host infections with increased domestic dog deworming coverage

**Fig 8:**
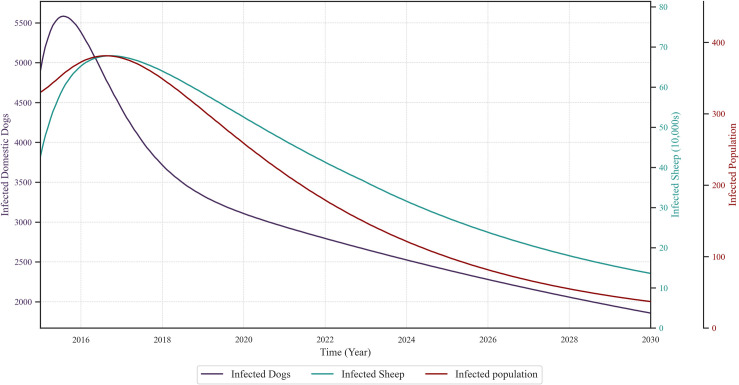
Simulation results for each host infection number after improving the deworming rate of domestic dogs.

To verify the simulation results, a scenario in which only the domestic dog deworming coverage was increased (Scenario 2–1) was simulated while all parameters for other control measures remained at their current levels (Current scenario). Trends in the number of infections among various hosts were observed. As shown in Fig 8, the peak in the number of infected domestic dogs occurred earlier than the peaks in the number of infected intermediate hosts and sheep; shortly thereafter, the number of infections in humans and sheep increased.

### 3.4. Optimization of control measures

The simulation results from scenario 1–1 and scenario 1–2 indicate that increasing and decreasing the vaccination coverage significantly affected the number of infected sheep, whereas the effect on the number of human cases was relatively small. The results from scenario 2–1 and scenario 2–2 show that the domestic dog deworming coverage should not be excessively increased. From the results of scenario 3–1 and scenario 3–2, it is evident that enhancing or weakening the sheep slaughtering management level leads to corresponding decreases and increases in the number of human cases and infected sheep, respectively. However, since both humans and sheep are intermediate hosts and sheep slaughter management directly affects dogs by reducing the number of cyst-containing organs they contact, the impact on humans and sheep is not as significant. The simulation results from scenario 4–1 and scenario 4–2 show that improving human publicity and education effectively raises awareness of preventive measures, reducing the risk of contact with tapeworm eggs, and is one of the most effective measures for preventing human echinococcosis.

**Fig 9:**
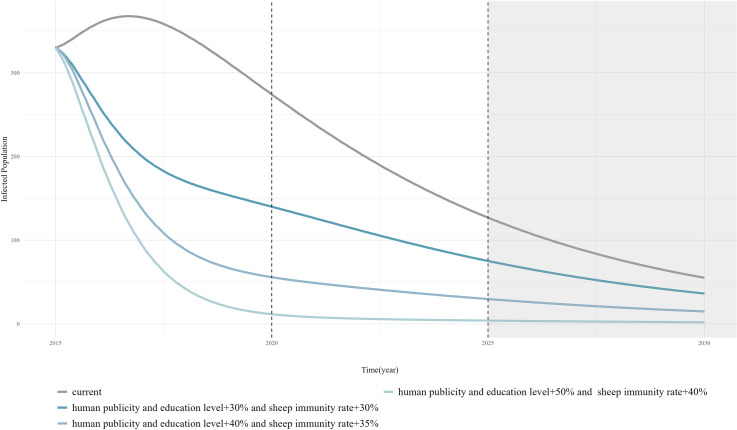
Simulation results for the number of human cases after optimization of key prevention and control measures for human echinococcosis.

**Fig 10:**
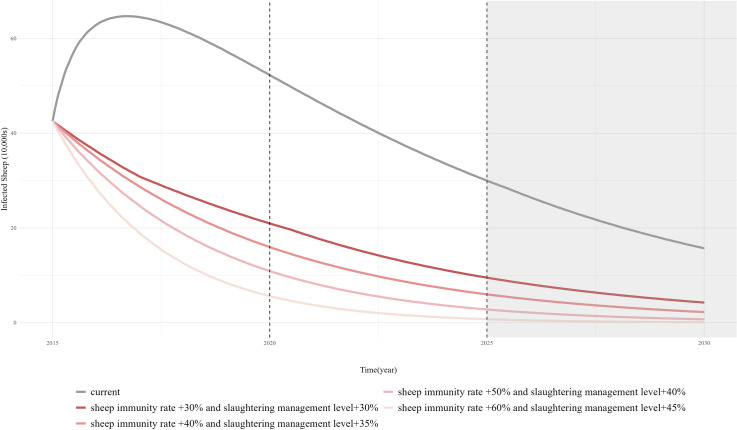
Simulation results for the number of infected sheep after optimization of key prevention and control for sheep echinococcosis.

Based on the simulation results, two key control measures should be comprehensively implemented for human echinococcosis (increasing health education level by 30% and vaccination coverage by 30%). Similarly, the most effective control measures for sheep echinococcosis are shown to be increasing the vaccination coverage by 30% and slaughter management level by 30%. Trends in the number of human cases and infected sheep were observed after applying these key control measures. The results, as shown in Figs 9 and 10, indicate a significant decrease in the number of human cases and infected sheep compared to the current control level (Current scenario), although the decrease tended to level off over time, indicating that echinococcosis may still persist. Therefore, based on this foundation, health education level were further increased by 10% increments, and the vaccination coverage was increased by 5% increments to observe the trend in the number of human cases. The vaccination coverage was increased in 10% increments and the sheep slaughtering management level was increased in 5% increments to observe trends in the number of infected sheep. The results showed that when health education level increased by 50% and the vaccination coverage increased by 40%, the number of human cases approached zero around 2026. When the vaccination coverage increases by 60% and the sheep slaughtering management level by 45%, the number of infected sheep approaches zero by 2027.

## 4. Discussion

### 4.1. Applicability of the SD model

Traditional mathematical models are widely used in echinococcosis control research and studies have shown that increasing the deworming rate in dogs can effectively reduce the prevalence of echinococcosis [28,29]. However, these models usually assume that the interactions within a system are linear and do not fully reflect the complex dynamics and nonlinear characteristics of infectious disease transmission. Using SD model simulation, this study revealed the nonlinear characteristics of echinococcosis transmission, particularly when the domestic dog deworming coverage is increased; the number of infected sheep and human cases first increases and then decreases in the short term, indicating that the transmission process is not simply linear. This pattern might be due to the increased deworming of domestic dogs resulting in a large amount of feces containing tapeworm eggs to be excreted. This temporarily increased the number of eggs in the environment and exposure of intermediate hosts to the eggs. Over time, however, many of the eggs die naturally, and with fewer infected domestic dogs, the number of eggs excreted decreases, thereby reducing human and sheep exposure. Rong et al. [23] also pointed out that there are complex feedback mechanisms in the disease transmission process and that merely increasing the domestic dog deworming coverage cannot effectively control the spread of echinococcosis. Through SD model analysis in this study, we validated its applicability in capturing the complex transmission mechanisms of echinococcosis. Atkinson et al. [30] reviewed previous studies on mathematical models and pointed out that traditional models may have deviations in long-term predictions, whereas SD models can more accurately reflect the nonlinear behavior of infectious diseases, thus providing more realistic control recommendations.

### 4.2. Key control measures for human echinococcosis and sheep echinococcosis

The results of the SD model indicated that enhancing educational campaigns, increasing the immunity rate of sheep flocks, and strengthening slaughter management are core strategies for controlling echinococcosis. First, educational campaigns are crucial for reducing infections in the human population. Craig et al. [17] pointed out that deworming dogs alone is insufficient for the effective control of echinococcosis, and must be combined with increased public awareness and other control measures to achieve significant results. Second, the vaccination of sheep flocks is vital for reducing the spread of echinococcosis within flocks. A study by Larrieu et al. [28] in Argentina showed that vaccinating sheep with the EG95 vaccine significantly lowered infection rates, thereby interrupting the disease transmission chain. Slaughter management is another indispensable control measure. Amarir et al. [31] noted that although deworming dogs played a role in reducing the spread of *Echinococcus granulosus*, the effect was limited. Only by combining sheep vaccination and strict slaughter management can the prevalence of echinococcosis be significantly reduced. Furthermore, Rong et al. [23] demonstrated that the effectiveness of canine deworming measures may be limited in environments with a high accumulation of worm eggs, especially in areas with many under wormed stray dogs. To effectively control echinococcosis, it is essential to integrate domestic dog deworming with centralized management of dog feces. However, owing to the low awareness of proper fecal disposal in endemic areas, the deworming rate should be maintained at current levels to prevent increased infection among intermediate hosts.

Through these measures, the SD model analysis in this study further validated that educational campaigns, sheep flock immunity, and slaughter management are core strategies for controlling echinococcosis. Cai et al. [7] also showed that these comprehensive intervention measures have brought significant economic benefits to the regions in which they have been implemented.

### 4.3. Quantitative optimization of control measures for human and sheep echinococcosis

Based on previous research, we utilized an SD model to quantitatively optimize comprehensive prevention and control measures for echinococcosis in humans and sheep. It quantifies the combined effects of these measures and provides specific optimization strategies. For instance, Zhang and Xiao [32] noted that relying solely on domestic dog deworming and stray dog management might not be sufficient for the long-term control of echinococcosis. Further quantitative analysis confirmed that a combination of public education and environmental management was an effective strategy for overall control. Specifically, increasing the level of public education by 50% and vaccination coverage by 40% are optimal for reducing human infection rates, whereas increasing vaccination coverage by 60% and slaughter management levels by 45% have the greatest effect on reducing sheep infection rates. This precise quantitative analysis provides a scientific basis for formulating targeted control measures. Using dynamic modeling, Rong et al. [23] showed that the effectiveness of a single measure is limited. Our quantitative analysis further optimizes the combination of multiple measures, confirms the best implementation ratios of comprehensive measures under resource-limited conditions, and offers more precise references for public health strategy formulation. Overall, this study optimizes the comprehensive prevention and control strategies for echinococcosis in the Gansu Province through quantitative optimization analysis, clarifies the optimal combination and implementation ratios of different control measures, extends the findings of previous research, and provides scientific guidance for the effective control of echinococcosis in the Gansu Province.

### 4.4. Limitations of the model and future research

#### 4.4.1. Extension of the SD model.

The current SD model primarily focuses on the transmission chain involving sheep as intermediate hosts and dogs as definitive hosts for echinococcosis. Future studies could extend the model by incorporating the impacts of other potential intermediate hosts (e.g., cattle, pigs) and definitive hosts (e.g., wolves, foxes) on disease transmission dynamics. Furthermore, the framework of this model could be adapted for investigating other vector-borne infections through structural modifications. By adjusting parameters related to host species, vector characteristics, and transmission pathways, this modeling approach may provide scientific foundations for developing control strategies against various infectious diseases. Such methodological extensions would enhance the model’s applicability in public health decision-making.

Furthermore, the transmission dynamics of echinococcosis are strongly influenced by environmental factors such as temperature and humidity, which exhibit distinct seasonal patterns. For example, warm and humid seasons may enhance the survival and dissemination of parasite eggs. The current model does not account for seasonal variations, potentially introducing biases in predictive outcomes. To address this limitation, future research could refine the model by incorporating region-specific seasonal parameters (e.g., monthly fluctuations in temperature and precipitation within the study area).

Additionally, the current model relies on fixed parameter values (Table 1) sourced from literature. While these values provide a practical basis for simulation, their inherent uncertainties stem from regional variations in disease transmission dynamics, measurement biases, or temporal fluctuations. Future extensions of this model should incorporate probabilistic approaches to quantify the impact of parameter uncertainty on intervention outcomes.

#### 4.4.2. Cross-regional transmission dynamics.

This study focused exclusively on echinococcosis transmission within Gansu Province, overlooking cross-regional transmission pathways. For instance, the inter-regional transportation of infected livestock (e.g., sheep) or canine movement across administrative boundaries could amplify disease spread. Future research should prioritize the development of cross-regional collaborative models to evaluate the effectiveness of coordinated prevention and control strategies across geographically connected areas.

#### 4.4.3 Economic and policy analysis.

Future research should further quantify the economic costs and benefits of different control measures to support evidence-based policymaking. For instance, it is essential to design culturally appropriate educational programs (e.g., bilingual health education initiatives). Vaccination coverage and slaughter management rely heavily on grassroots veterinary personnel, necessitating strengthened development of primary-level disease prevention infrastructure. Furthermore, indiscriminate increases in deworming rates may temporarily exacerbate environmental contamination with parasite eggs, underscoring the need for concurrent implementation of integrated measures including proper fecal waste management systems. Analyzing the cost-effectiveness ratios of health education, vaccination programs, and slaughter management could optimize resource allocation. Such analyses would offer policymakers comprehensive insights for prioritizing interventions under budget constraints.

## 5. Conclusion

This study analyzed the annual incidence data of sheep and human echinococcosis in Gansu Province from 2015 to 2022 using an SD model to simulate the disease situation and infection status. The model indicates that control measures focused on enhancing health education level and increasing the vaccination coverage are significantly effective in reducing the number of human cases and infected sheep, respectively. Furthermore, maintaining the current level of domestic dog deworming is sufficient; however, after improving the effective treatment of dog feces, the dog deworming rate can be appropriately increased. The findings of this study provide strategic recommendations for the control of echinococcosis in the Gansu Province and offer new insights and methods for the future control of other zoonotic diseases. By focusing on these key control measures, significant progress can be made in reducing the incidence of echinococcosis and ultimately improving public health outcomes in endemic regions.

## Supporting information

S1 Text Causal feedback loop diagram and analysis of echinococcosis transmission dynamics. (DOCX)

S2 Text System dynamics model equations for echinococcosis transmission. (DOCX)

S3 Text The calculation of baseline parameters in Table 2. (DOCX)

## References

[1] Wang L-Y, Qin M, Gavotte L, Wu W-P, Cheng X, Lei J-X, et al. Societal drivers of human echinococcosis in China. Parasit Vectors. 2022;15(1):385. doi: 10.1186/s13071-022-05480-8 36271415 PMC9587573

[2] Craig PS, McManus DP, Lightowlers MW, Chabalgoity JA, Garcia HH, Gavidia CM, et al. Prevention and control of cystic echinococcosis. Lancet Infect Dis. 2007;7(6):385–94. doi: 10.1016/S1473-3099(07)70134-2 17521591

[3] Wang L-Y, Qin M, Liu Z-H, Wu W-P, Xiao N, Zhou X-N, et al. Prevalence and spatial distribution characteristics of human echinococcosis in China. PLoS Negl Trop Dis. 2021;15(12):e0009996. doi: 10.1371/journal.pntd.0009996 34962928 PMC8789093

[4] Kui Y, Liu B, Wang X, Xue C, Xue J, Zhang Y, et al. Epidemiological Characteristics of Echinococcosis in Non-Endemic PLADs - China, 2017-2020. China CDC Wkly. 2021;3(51):1084–8. doi: 10.46234/ccdcw2021.262 34938586 PMC8688752

[5] Luo A, Wang H, Li J-Q, Wu H-S, Yang F, Fang P-Q. Epidemic factors and control of hepatic echinococcosis in Qinghai province. J Huazhong Univ Sci Technolog Med Sci. 2014;34(1):142–5. doi: 10.1007/s11596-014-1246-8 24496694

[6] Coordinating Office of the National Survey on the Important Human Parasitic Diseases. A national survey on current status of the important parasitic diseases in human population. Zhongguo Ji Sheng Chong Xue Yu Ji Sheng Chong Bing Za Zhi. 2005;23(5 Suppl):332–40. 16562464

[7] Cai J, Yang K, Chen Q, Zhao Q, Li J, Wang S, et al. The impact of echinococcosis interventions on economic outcomes in Qinghai Province of China: Evidence from county-level panel data. Front Vet Sci. 2023;10:1068259. doi: 10.3389/fvets.2023.1068259 37008365 PMC10063884

[8] Thompson RCA. Biology and Systematics of Echinococcus. Adv Parasitol. 2017;95:65–109. doi: 10.1016/bs.apar.2016.07.001 28131366

[9] Torgerson PR, Devleesschauwer B, Praet N, Speybroeck N, Willingham AL, Kasuga F, et al. World Health Organization Estimates of the Global and Regional Disease Burden of 11 Foodborne Parasitic Diseases, 2010: A Data Synthesis. PLoS Med. 2015;12(12):e1001920. doi: 10.1371/journal.pmed.1001920 26633705 PMC4668834

[10] Han S, Kui Y, Xue C, Zhang Y, Zhang B, Li Q, et al. The endemic status of echinococcosis in China from 2004 to 2020. Chinese Journal of Parasitology and Parasitic Diseases. 2022;40(4):475–80. doi: 10.12140/j.issn.1000-7423.2022.04.009

[11] Yu Q, Xiao N, Han S, Tian T, Zhou X-N. Progress on the national echinococcosis control programme in China: analysis of humans and dogs population intervention during 2004-2014. Infect Dis Poverty. 2020;9(1):137. doi: 10.1186/s40249-020-00747-7 33008476 PMC7532088

[12] Taratuto AL, Venturiello SM. Echinococcosis. Brain Pathol. 1997;7(1):673–9. doi: 10.1111/j.1750-3639.1997.tb01082.x 9034573 PMC8098587

[13] Yan W-L, Meng J-X, Li X-M, Zhao J-P, Zhang M, Wang X-Y, et al. Global Prevalence of Echinococcosis in Goats: A Systematic Review and Meta-Analysis. Foodborne Pathog Dis. 2022;19(10):675–85. doi: 10.1089/fpd.2022.0030 36036962

[14] Jensenius M, Mørch K, Yaqub S, Halvorsen DS, Reims HM, Björk IG, et al. Alveolar echinococcosis. Tidsskr Nor Laegeforen. 2024;144(10):10.4045/tidsskr.24.0121. doi: 10.4045/tidsskr.24.0121 39254012

[15] Richardson GP. Reflections on the foundations of system dynamics. System Dynamics Review. 2011;27(3):219–43. doi: 10.1002/sdr.462

[16] Homer JB, Hirsch GB. System dynamics modeling for public health: background and opportunities. Am J Public Health. 2006;96(3):452–8. doi: 10.2105/AJPH.2005.062059 16449591 PMC1470525

[17] Craig PS, Hegglin D, Lightowlers MW, Torgerson PR, Wang Q. Echinococcosis: Control and Prevention. Adv Parasitol. 2017;96:55–158. doi: 10.1016/bs.apar.2016.09.002 28212791

[18] He Y, Cui Q, Hu Z. Modeling and analysis of the transmission dynamics of cystic echinococcosis: Effects of increasing the number of sheep. Math Biosci Eng. 2023;20(8):14596–615. doi: 10.3934/mbe.2023653 37679150

[19] Fu M-H, Wang X, Han S, Guan Y-Y, Bergquist R, Wu W-P. Advances in research on echinococcoses epidemiology in China. Acta Trop. 2021;219:105921. doi: 10.1016/j.actatropica.2021.105921 33878307

[20] Yan S, Wang D, Zhang J, Mo X, Feng Y, Duan L, et al. Epidemiological survey of human echinococcosis in east Gansu, China. Sci Rep. 2021;11(1):6373. doi: 10.1038/s41598-021-85843-w 33737680 PMC7973574

[21] Wang Q, Huang Y, Huang L, Yu W, He W, Zhong B, et al. Review of risk factors for human echinococcosis prevalence on the Qinghai-Tibet Plateau, China: a prospective for control options. Infect Dis Poverty. 2014;3(1):3. doi: 10.1186/2049-9957-3-3 24475907 PMC3910240

[22] Bala BK. System dynamics modelling and simulation of biogas production systems. Renewable Energy. 1991;1(5–6):723–8. doi: 10.1016/0960-1481(91)90019-l

[23] Rong X, Fan M, Zhu H, Zheng Y. Dynamic modeling and optimal control of cystic echinococcosis. Infect Dis Poverty. 2021;10(1):38. doi: 10.1186/s40249-021-00807-6 33762009 PMC7992812

[24] Torgerson PR. Mathematical models for the control of cystic echinococcosis. Parasitol Int. 2006;55 Suppl:S253-8. doi: 10.1016/j.parint.2005.11.037 16364679

[25] Wang K, Zhang X, Jin Z, Ma H, Teng Z, Wang L. Modeling and analysis of the transmission of Echinococcosis with application to Xinjiang Uygur Autonomous Region of China. J Theor Biol. 2013;333:78–90. doi: 10.1016/j.jtbi.2013.04.020 23669505

[26] Lightowlers MW, Colebrook AL, Gauci CG, Gauci SM, Kyngdon CT, Monkhouse JL, et al. Vaccination against cestode parasites: anti-helminth vaccines that work and why. Vet Parasitol. 2003;115(2):83–123. doi: 10.1016/s0304-4017(03)00202-4 12878418

[27] Gutiérrez ML, Di Federico G, Dale JA, Minoia JM, Corrales CD, Schaiquevich P, et al. Pharmacokinetics of a novel spot-on formulation of praziquantel for dogs. Vet Parasitol. 2017;239:46–9. doi: 10.1016/j.vetpar.2017.04.022 28495196

[28] Larrieu E, Gavidia CM, Lightowlers MW. Control of cystic echinococcosis: Background and prospects. Zoonoses Public Health. 2019;66(8):889–99. doi: 10.1111/zph.12649 31529690

[29] Yang R, Zhao J, Yan Y. Stability Analysis and Optimal Control Strategies of an Echinococcosis Transmission Model. Comput Math Methods Med. 2022;2022:6154866. doi: 10.1155/2022/6154866 35651923 PMC9151005

[30] Atkinson J-AM, Williams GM, Yakob L, Clements ACA, Barnes TS, McManus DP, et al. Synthesising 30 years of mathematical modelling of Echinococcus transmission. PLoS Negl Trop Dis. 2013;7(8):e2386. doi: 10.1371/journal.pntd.0002386 24009786 PMC3757076

[31] Amarir F, Rhalem A, Sadak A, Raes M, Oukessou M, Saadi A, et al. Control of cystic echinococcosis in the Middle Atlas, Morocco: Field evaluation of the EG95 vaccine in sheep and cesticide treatment in dogs. PLoS Negl Trop Dis. 2021;15(3):e0009253. doi: 10.1371/journal.pntd.0009253 33684115 PMC7971873

[32] Zhang Y, Xiao Y. Modeling and analyzing the effects of fixed‐time intervention on transmission dynamics of echinococcosis in Qinghai province. Math Methods in App Sciences. 2021;44(6):4276–96. doi: 10.1002/mma.7029

