# Supplementary material for: Optimization of echinococcosis control measures based on system dynamics
Source: PLoS Comput Biol. 2025 Sep 30;21(9):e1013186. doi: 10.1371/journal.pcbi.1013186 (PMC12483238; doi:10.1371/journal.pcbi.1013186)
Supplement: S1 Text — (DOCX) [file pcbi.1013186.s001.docx]

**S1 Text – Causal feedback loop diagram and analysis of echinococcosis transmission dynamics**

The transmission of echinococcosis occurs through the completion of Echinococcus tapeworms' life cycle involving intermediate hosts (humans, sheep, etc.) and definitive hosts (canines). Interventions including deworming domestic dogs, vaccinating sheep populations, implementing post-slaughter management of livestock, and conducting public health education campaigns can effectively reduce disease prevalence. Based on these mechanisms, we constructed a causal feedback loop diagram for echinococcosis transmission (S1 Fig.). In this diagram, nodes represent various factors within the transmission system, while arrows indicate direct influence relationships between these factors. The primary feedback loops identified include:

1.1 Positive feedback loops

1.1.1 Positive feedback loop on “Dog infection rate”

Infected dogs↑ → Environmental egg quantity↑ → Sheep infection rate↑ → Infected sheep↑ → Hydatid cysts↑ → Dog infection rate↑

1.1.2 Positive feedback loop on “Sheep infection rate”

Environmental egg quantity↑ → Sheep infection rate↑ → Infected sheep↑ → Hydatid cysts↑ → Dog infection rate↑ → Infected dogs↑ → Environmental egg quantity↑ → Sheep infection rate↑

1.1.3 Positive feedback loop on “Human infection rate”

Environmental egg quantity↑ → Human infection rate↑ → Exposed human population↑ → Infected population↑

1.2 Negative feedback loops

1.2.1 Negative feedback loop on “Vaccination coverage”

Vaccination coverage↑ → Sheep infection rate↓ → Hydatid cysts↓ → Dog infection rate↓ →Infected dogs↓ → Environmental egg quantity↓ → Sheep infection rate↓

1.2.2 Negative feedback loop on “Dog deworming coverage”

Dog deworming coverage↑ → Infected dogs↓ → Environmental egg quantity↓ → Sheep infection rate↓ → Hydatid cysts↓ → Dog infection rate↓

1.2.3 Negative feedback loop on “Health education level”

Health education level↑ → Human infection rate↓ → Exposed human population↓ → Infected population↓

1.2.4 Negative feedback loop on “Slaughter management level”

Slaughter management level↑ → Hydatid cysts↓ → Dog infection rate↓ → Infected dogs↓ → Environmental egg quantity↓ → Sheep infection rate↓


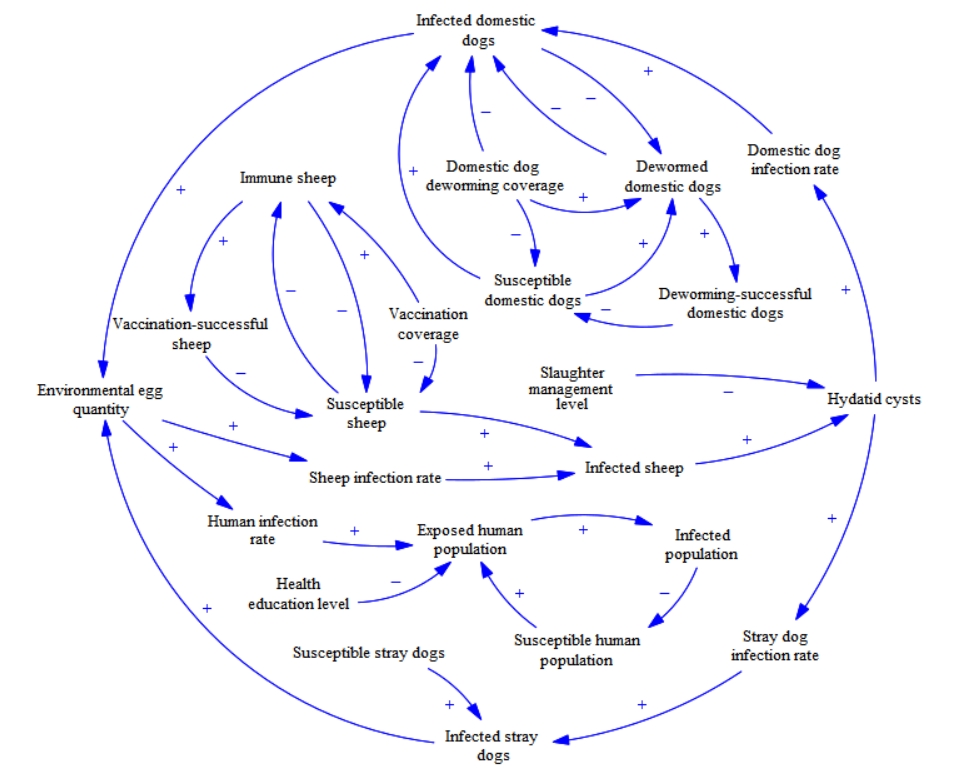


Fig 1. Diagram of the causal feedback loop for echinococcosis transmission
