# Supplementary material for: Optimization of echinococcosis control measures based on system dynamics
Source: PLoS Comput Biol. 2025 Sep 30;21(9):e1013186. doi: 10.1371/journal.pcbi.1013186 (PMC12483238; doi:10.1371/journal.pcbi.1013186)
Supplement: S2 Text — (DOCX) [file pcbi.1013186.s002.docx]

**S2 Text – System Dynamics model equations for echinococcosis transmission**

1. Susceptible sheep population = INTEG(Annual new sheep recruitment + Vaccination failures + Waning vaccine immunity - New infected sheep - Susceptible sheep removal - Sheep vaccination implementation)
2. Infected sheep population = INTEG(New infected sheep - Infected sheep removal)
3. Immune sheep population = INTEG(Sheep vaccination implementation - Vaccination failures - Vaccination-successful sheep - Immune sheep removal)
4. Vaccination-successful sheep population = INTEG(Vaccination-successful sheep - Waning vaccine immunity - Vaccination-successful removal)
5. Annual new sheep recruitment = Susceptible sheep population × Sheep birth rate
6. Susceptible sheep removal = Susceptible sheep population × Sheep mortality rate + Susceptible sheep population × Slaughter rate
7. New infected sheep = Susceptible sheep population × Sheep infection rate
8. Infected sheep removal = Infected sheep population × Slaughter rate + Infected sheep population × Sheep mortality rate
9. Sheep vaccination implementation = Susceptible sheep population × Vaccination coverage
10. Immune sheep removal = Immune sheep population × Sheep mortality rate + Immune sheep population × Slaughter rate
11. Vaccination-successful sheep = Immune sheep population × Vaccine efficacy rate
12. Vaccination-successful sheep removal = Vaccination-successful sheep population × Slaughter rate + Vaccination-successful sheep population × Sheep mortality rate
13. Vaccination failures = Immune sheep population × Vaccine failure rate
14. Waning vaccine immunity= Vaccination-successful sheep population × Waning vaccine immunity probability
15. Effective sheep-egg contact = Environmental egg quantity × Sheep-egg contact coefficient
16. Discarded offal = Infected sheep population × Slaughter rate × (1 - Slaughter management level)
17. Total hydatid cysts = Discarded offal × Hydatid cyst burden per offal
18. Sheep infection rate = IF THEN ELSE(Effective sheep-egg contact >= 500, 1, Effective sheep-egg contact × 1/500)
19. Susceptible human population = INTEG(Annual human births + Recovery cases - New exposed cases - Susceptible human removal)
20. Exposed human population = INTEG(New exposed cases - Clinical cases - Exposed removal)
21. Infected population = INTEG(Clinical cases - Recovery cases - Clinical removal)
22. Annual human births = Susceptible human population × Human birth rate
23. Susceptible human removal = Susceptible human population × Background human mortality
24. New exposed cases = Susceptible human population × Human infection rate
25. Exposed removal = Exposed human population × Background human mortality
26. Clinical cases = Exposed human population × Disease progression rate
27. Infected population removal = Infected population × Case fatality rate
28. Recovery cases = Infected population × Recovery rate
29. Effective human-egg contact = Environmental egg quantity × Human-egg contact coefficient × (1 - Health education level)
30. Human infection rate = IF THEN ELSE(Effective human-egg contact >= 500, 1, Effective human-egg contact × 1/500)
31. Susceptible domestic dogs = INTEG(Annual domestic dog recruitment + Loss of deworming efficacy - New infected domestic dogs - Susceptible domestic dog deworming - Susceptible domestic dog removal)
32. Infected domestic dogs = INTEG(New infected domestic dogs + Deworming failures - Infected domestic dog deworming - Infected domestic dog removal)
33. Dewormed domestic dogs = INTEG(Susceptible domestic dog deworming + Infected domestic dog deworming - Deworming successes - Deworming failures - Dewormed domestic dog removal)
34. Deworming-successful domestic dogs = INTEG(Deworming successes - Loss of deworming efficacy - Deworming-successful domestic dogs removal)
35. Susceptible stray dogs = INTEG(Annual stray dog recruitment - New infected stray dogs - Susceptible stray dogs removal)
36. Infected stray dogs = INTEG(New infected stray dogs - Infected stray dogs removal)
37. Environmental egg quantity = INTEG(Eggs excreted per dog - Eggs decay count)
38. Annual domestic dog recruitment = Susceptible domestic dogs × Domestic dog birth rate
39. Annual stray dog recruitment = Susceptible stray dogs × Stray dog birth rate
40. Susceptible domestic dogs removal = Susceptible domestic dogs × Domestic dog mortality
41. Susceptible stray dogs removal = Susceptible stray dogs × Stray dog control rate + Susceptible stray dogs × Stray dog mortality
42. New infected domestic dogs = Susceptible domestic dogs × Domestic dog infection rate
43. New infected stray dogs = Susceptible stray dogs × Stray dog infection rate
44. Infected domestic dogs removal = Infected domestic dogs × Domestic dog mortality
45. Infected stray dogs removal = Infected stray dogs × Stray dog control rate + Infected stray dogs × Stray dog mortality
46. Susceptible domestic dog deworming = Susceptible domestic dogs × Domestic dog deworming coverage
47. Infected domestic dog deworming = Infected domestic dogs × Domestic dog deworming coverage
48. Deworming failures = Dewormed domestic dogs × Deworming failure probability
49. Deworming successes = Dewormed domestic dogs × Deworming efficacy rate
50. Loss of deworming efficacy = Deworming-successful domestic dogs × Deworming efficacy loss rate
51. Deworming-successful domestic dogs removal = Deworming-successful domestic dogs × Domestic dog mortality
52. Dewormed domestic dog removal = Dewormed domestic dogs × Domestic dog mortality
53. Eggs excreted per dog = Infected domestic dogs × Dog defecation frequency × Eggs per defecation + Infected stray dogs × Dog defecation frequency × Eggs per defecation
54. Eggs decay count = Environmental egg quantity × Natural decay rate
55. Effective dog-cyst contact = Total hydatid cysts × Dog-cyst contact coefficient
56. Domestic dog infection rate = IF THEN ELSE(Effective dog-cyst contact >= 100, 1, Effective dog-cyst contact × 1/100)
57. Stray dog infection rate = IF THEN ELSE(Effective dog-cyst contact >= 100, 1, Effective dog-cyst contact × 1/100)
