# Supplementary material for: Optimization of echinococcosis control measures based on system dynamics
Source: PLoS Comput Biol. 2025 Sep 30;21(9):e1013186. doi: 10.1371/journal.pcbi.1013186 (PMC12483238; doi:10.1371/journal.pcbi.1013186)
Supplement: S3 Text — (DOCX) [file pcbi.1013186.s003.docx]

**S3 Text - The calculation of baseline parameters in Table 2**

1. **Overall Situation of the Questionnaire**

A total of 550 questionnaires were distributed in three echinococcosis-endemic counties, with 180 in Maqu County, 200 in Tianzhu County, and 170 in Subei County. In total, 501 questionnaires were recovered, with 162 in Maqu County, 186 in Tianzhu County, and 153 in Subei County. The overall recovery rate of the questionnaires was 91.1%. Among them, 462 questionnaires were valid, with 149 in Maqu County, 166 in Tianzhu County, and 147 in Subei County. The overall validity rate of the questionnaires reached 92.2%.

1. **Calculation Sources and Methods of the Four Parameters**

**Overall Premise Explanation:** The survey was conducted at the household level, with all households in pastoral areas engaged in sheep raising. Data for each parameter were collected and analyzed based on household-level statistics.

- 1. **Vaccination coverage**

**Data Source:** Household-level statistics on sheep vaccination status, including vaccination rates and post-vaccination antibody detection.

**Calculation Method:** Vaccination coverage was determined by the proportion of households reporting complete sheep vaccination. The formula used is:

$$\text{V}\text{=}\frac{\text{Number of households with vaccinated sheep}}{\text{Total number of households}}$$

Where *V* represents the vaccination coverage. The baseline value of *V* was determined through household questionnaire surveys and statistical analysis, and the final baseline value was confirmed as 0.1996.

- 1. **Domestic dog deworming coverage**

**Data Source:** Deworming practices were recorded for households keeping domestic dogs (63.42% of total households)

**Calculation Method:** Deworming coverage was calculated as the proportion of dog-owning households implementing regular deworming. The formula is:

$$\text{D}\text{=}\frac{\text{Number of households with regularly dewormed domestic dogs}}{\text{Total }\text{dog}\text{-owning}\text{ households}}$$

Where *D* denotes domestic dog deworming coverage. The baseline value of 0.6758 was established based on the proportion of households reporting regular deworming.

- 1. **Slaughter management level**

**Data Source:** Household practices for post-slaughter organ disposal, particularly rates of harmless treatment (e.g., deep burial or incineration).

**Calculation Method:** Slaughter management level was assessed by the proportion of households using harmless disposal methods for sheep organs. The formula is:

$$\text{S}\text{=}\frac{\text{Households with harmless organ disposal}}{\text{Total households conducting sheep slaughter}}$$

With *S* representing slaughter management level, the baseline value of 0.5152 was determined by integrating household disposal data and expert evaluations of treatment effectiveness in questionnaire surveys.

- 1. **Health education level**

**Data Source:** Household-level awareness of echinococcosis prevention, including knowledge of transmission routes and control measures.

**Calculation Method:** Health education level was measured by the average awareness rate of core prevention knowledge among household members. The formula is:

$$\text{H}\text{=}\frac{\text{Households with correct prevention knowledge}}{\text{Total surveyed households}}$$

Where *H* represents health education level. The baseline value of 0.6542 was derived from the overall awareness rate of echinococcosis prevention knowledge across households.
